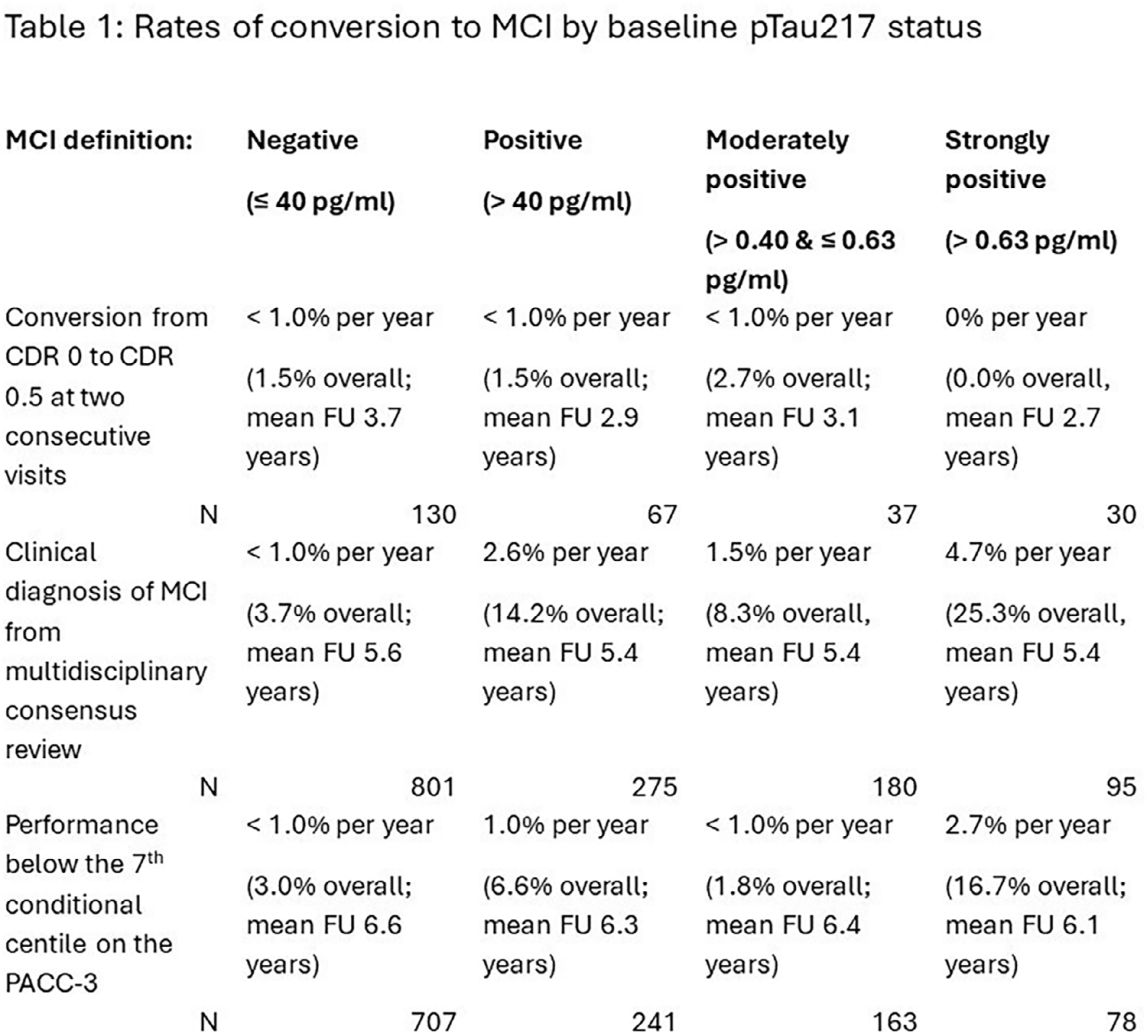# Plasma pTau217 levels and conversion to MCI in the Wisconsin Registry for Alzheimer's Prevention and the Wisconsin ADRC

**DOI:** 10.1002/alz70856_105805

**Published:** 2026-01-07

**Authors:** Julie E. Oomens, Rachael E. Wilson, Ramiro Eduardo Rea Reyes, Erin M. Jonaitis, Nathaniel A. Chin, Henrik Zetterberg, Sterling C Johnson

**Affiliations:** ^1^ Wisconsin Alzheimer's Disease Research Center, University of Wisconsin School of Medicine and Public Health, Madison, WI, USA; ^2^ Wisconsin Alzheimer's Disease Research Center, University of Wisconsin‐Madison, School of Medicine and Public Health, Madison, WI, USA; ^3^ Wisconsin Alzheimer's Institute, University of Wisconsin School of Medicine and Public Health, Madison, WI, USA; ^4^ Hong Kong Center for Neurodegenerative Diseases, Hong Kong, Science Park, China; ^5^ Department of Psychiatry and Neurochemistry, Institute of Neuroscience and Physiology, the Sahlgrenska Academy, University of Gothenburg, Molndal, Sweden; ^6^ Department of Neurodegenerative Disease, UCL Institute of Neurology, London, United Kingdom; ^7^ UK Dementia Research Institute, University College London, London, United Kingdom; ^8^ Clinical Neurochemistry Laboratory, Sahlgrenska University Hospital, Mölndal, Västra Götaland län, Sweden; ^9^ Wisconsin Alzheimer's Institute, University of Wisconsin School of Medicine and Public Health, Madison, WI, USA; ^10^ Wisconsin Alzheimer's Disease Research Center, University of Wisconsin School of Medicine and Public Health, Madison, WI, USA

## Abstract

**Background:**

As the evidence base supporting the accuracy and reliability of plasma pTau217 assays continues to grow, attention has shifted towards establishing their diagnostic and clinical utility. The aim of the current study was to examine the association between plasma pTau217 levels and conversion to MCI in a preclinical cohort.

**Method:**

We selected 1943 participants from the Wisconsin Registry for Alzheimer's Prevention and the Wisconsin ADRC for whom Quanterix HD‐X plasma pTau217 data was available. Participants were classified as being plasma pTau217 negative (≤ 40 pg/ml, *n* = 1402) or positive (> 40 pg/ml, *n* = 541), with the positive group also being subdivided into moderately positive (> 0.40 & ≤ 0.63 pg/ml, *n* = 308) and strongly positive (> 0.63 pg/ml, *n* = 233). Incident MCI was defined using varying levels of stringency and definitions included (1) conversion from CDR 0 to CDR 0.5 at two consecutive visits (*n* = 197, median 3 visits, mean FU 3.4 years; definition 1), (2) a clinical diagnosis of MCI from multidisciplinary consensus review (*n* = 1076, median 5 visits, mean FU 5.6 years; definition 2) and (3) performance below the 7^th^ conditional centile on the PACC‐3 for two consecutive visits (*n* = 948, median 3 visits, mean FU 6.6 years; definition 3).

**Result:**

The mean age of the included participants was 63 years (SD8.1). 68% of participants were female, 35% of participants were APOE e4 carrier and 83% of participants were non‐Hispanic white. Rates of conversion in the total cohort were < 1% per year (1.5% overall; average time to conversion 5.1 years) for definition 1, 1.1% per year (6.4% overall; average time to conversion 7.3 years) for definition 2 and <1.0% per year (3.9% overall; average time to conversion 5.7 years) for definition 3. Rates of conversion in the subset of pTau217 positive participants ranged from <1.0% to 4.7% per year (Table 1). Those with strongly positive plasma levels showed the highest rates of conversion (Table 1).

**Conclusion:**

Rates of conversion to MCI in this largely preclinical cohort ranged from < 1% to 1.9% per year in the total cohort and from <1% to 4.7% per year in the subset of participants that were plasma pTau217 positive.